# Spring and Late Summer Phytoplankton Biomass Impact on the Coastal Sediment Microbial Community Structure

**DOI:** 10.1007/s00248-018-1229-6

**Published:** 2018-07-17

**Authors:** Elias Broman, Lingni Li, Jimmy Fridlund, Fredrik Svensson, Catherine Legrand, Mark Dopson

**Affiliations:** Centre for Ecology and Evolution in Microbial Model Systems (EEMiS), Department of Biology and Environmental Science, Linnaeus University, 39182 Kalmar, Sweden

**Keywords:** 16S rRNA, Cyanobacteria, Diatom, Phytoplankton, Algae, Bloom, Sediment

## Abstract

Two annual Baltic Sea phytoplankton blooms occur in spring and summer. The bloom intensity is determined by nutrient concentrations in the water, while the period depends on weather conditions. During the course of the bloom, dead cells sink to the sediment where their degradation consumes oxygen to create hypoxic zones (< 2 mg/L dissolved oxygen). These zones prevent the establishment of benthic communities and may result in fish mortality. The aim of the study was to determine how the spring and autumn sediment chemistry and microbial community composition changed due to degradation of diatom or cyanobacterial biomass, respectively. Results from incubation of sediment cores showed some typical anaerobic microbial processes after biomass addition such as a decrease in NO_2_^−^ + NO_3_^−^ in the sediment surface (0–1 cm) and iron in the underlying layer (1–2 cm). In addition, an increase in NO_2_^−^ + NO_3_^−^ was observed in the overlying benthic water in all amended and control incubations. The combination of NO_2_^−^ + NO_3_^−^ diffusion plus nitrification could not account for this increase. Based on 16S rRNA gene sequences, the addition of cyanobacterial biomass during autumn caused a large increase in ferrous iron-oxidizing archaea while diatom biomass amendment during spring caused minor changes in the microbial community. Considering that OTUs sharing lineages with acidophilic microorganisms had a high relative abundance during autumn, it was suggested that specific niches developed in sediment microenvironments. These findings highlight the importance of nitrogen cycling and early microbial community changes in the sediment due to sinking phytoplankton before potential hypoxia occurs.

**Electronic supplementary material:**

The online version of this article (10.1007/s00248-018-1229-6) contains supplementary material, which is available to authorized users.

## Introduction

Seasonal hypoxia in coastal systems with slow water renewal is increasing worldwide as a result of nutrient discharge and rising water temperatures due to climate change [1]. During the transition of oxic to hypoxic (< 2 mg/L O_2_) and anoxic conditions in the water/sediment, hydrogen sulfide diffuses out of the sediment and accumulates in the bottom water. This creates toxic conditions for benthic life [2] and these areas are commonly referred to as “dead zones” [3]. These zones have increased tenfold over the last century, including in coastal bottom-waters during the last 50 years [4, 5]. The negative impact of dead zones extends to all trophic levels of coastal ecosystems including benthic and pelagic communities, but also on biodiversity and ecosystem services [6–10].

During the last 150 years, the Baltic Sea region has undergone drastic changes including population growth and its associated increase in agriculture and industry. While models link rising temperatures to the expansion of hypoxia in the Baltic Sea as far back as 1000 years [11], the primary driving factor of increasing and widespread hypoxic conditions is eutrophication [4]. Since the turn of the twentieth century, nutrient pools (nitrogen and phosphorus) have increased two- to threefold in the Gulf of Finland and the Baltic proper, while annual production of organic carbon perhaps increased even more [12, 13]. Compared to worldwide pelagic phytoplankton bloom patterns, the peculiarity of the Baltic Sea resides in a large winter-spring bloom with a dominance of diatoms and dinoflagellates and a summer bloom composed of N_2_-fixing cyanobacteria [14]. During the last 50 years, the increase in primary production in the Baltic pelagic system [15] has been driven by a shift from spring to summer primary production [13] with consequences for biogeochemical cycling. In the Baltic proper, changes in nutrient pools and the depletion of oxygen in bottom waters/sediments result in high phosphorus availability from sediment out-flux [16]. As nitrogen concentrations limit the spring bloom, more phosphorus is available for the summer bloom. Records for the past 8000 years from Baltic Sea sediment cores suggest that hypoxia sustains cyanobacterial blooms, most likely because of enhanced recycling of phosphorus in low oxygen conditions [17]. However, how the Baltic Sea sediment microbial community alters as a result of primary production during spring and summer blooms is largely unknown.

Climate warming and a decrease in salinity affect both the extent and composition of the blooms [18, 19]. The fate of the blooms is highly specific to the phytoplankton groups that differently affect pelagic nutrient cycling and benthic oxygen demand [20]. During summer and stratified conditions, pelagic nutrient recycling is likely higher than during the spring bloom, due to e.g. the accumulation of nitrogen fixating cyanobacteria (diazotrophs) capable of buoyancy regulation [21], picocyanobacteria uptake of N exuded by diazotrophs in conjunction with zooplankton grazing on pico- and diazotrophic cyanobacteria [22]. However, a significant amount of decaying phytoplankton sinks to the sediment increasing the benthic oxygen demand of aerobic microbes [3, 12, 16, 23, 24]. The resulting increase in sediment anoxia traps organic carbon due to slower degradation rates compared to oxygen rich sediment [25, 26]. This in turn leads to a decrease in the carbon budget and benthic-pelagic exchange [6]. As hypoxia progresses in the Baltic Sea, the dynamics of microbial communities in hypoxic water in relation to seasonal phytoplankton blooms have just begun to be explored with the use of high throughput sequencing [27].

In addition to studies on hypoxic waters, several investigations around the world have analyzed microbial community changes in sediment due to phytoplankton degradation. For example, *Flavobacteria* increased during spring blooms in the Western English Channel [28]. Sediment from the Gulf of Mexico amended with *Spirulina* phytoplankton-biomass contained *Gamma-* and *Deltaproteobacteria*, *Planctomycetes*, *Verrumicrobia*, and *Actinobacteria* [29]. Finally, addition of phytoflagellates stimulated *Alphaproteobacteria* while diatoms promoted a wide variety of bacterial taxa in coastal marine sediment from Brazil [30]. A few studies report alterations of Baltic Sea microbial communities due to phytoplankton addition or oxygen availability. For example, cyanobacterial biomass degradation increased the release of fermentation products in the surface of anoxic tidal-flat sediment [31]. Differences in *Proteobacteria* classes in surficial sediment can depend on oxygen availability [32] while the structure and function of microbial communities can be partly explained by the chemistry and stratification of the water column along a Landsort Deep sediment redox gradient [33]. The ability to survive oxic to hypoxic transitions is paramount for microbial communities to succeed in these environments. However, the changes in sediment microbial communities in relation to the degradation of different phytoplankton taxa/blooms remain largely unexplored.

In this study, two microbial communities in the sediment surface were studied: (i) during spring bloom conditions and (ii) during autumn degradation of the late summer bloom. Small amounts of diatom biomass during spring or cyanobacterial biomass during autumn were added to intact sediment cores from an all-year oxic site in a Baltic Sea coastal bay. We aimed to characterize the identities and diversity of the in situ sediment microbial community due to degradation of phytoplankton organic matter (OM). This knowledge is important as dead zones drastically limit possibilities for macrobiotic life and the exchange of nutrients between the pelagic and bottom water.

## Materials and Methods

### Autumn Sampling and Preparation of Cyanobacteria Biomass

Baltic Sea sediment cores were sampled on the 9 October 2014 at a coastal site near the town Loftahammar, Sweden (WGS 84 coordinates 57 53.214, 16 35.934). Based upon previous oxygen measurements of both the sediment and bottom water (water depth of 6.5 m) in November 2013, May 2014, October 2014, November 2014, and April 2015, we infer that this site is oxic all-year round [34]. Sediment with overlying bottom water was sampled using a gravity corer in combination with 60 cm long polymethylmethacrylate tubes (inner diameter 7 cm). Twenty sediment cores were collected with an average sediment height of 18.8 cm. Three sediment cores were sacrificed in the field and 50 mL of the bottom water transferred to sterile tubes for subsequent DNA extraction and 16 mL transferred to acid washed tubes to measure water chemistry. From these cores, the top 1 cm sediment was sliced into sterile tubes from which 1 mL sediment was aseptically transferred into pre-weighed microcentrifuge tubes for OM (% wt) determination; 10 to 16 mL transferred into tubes to measure pore-water chemistry; and the remaining sediment was used for DNA extraction (all procedures described below). All tubes were kept on ice during transport. The remaining sediment cores were transported back to the laboratory for incubation. In the laboratory, tubes for water chemistry were frozen at − 20 °C until analysis while water for DNA extraction was filtered with a 0.2-μm filter (Supor-200, PALL Corporation). The filters were aseptically transferred into sterile tubes and stored in − 80 °C until DNA extraction.

Filamentous cyanobacterial strains isolated from Baltic Sea summer blooms were obtained from the Kalmar Algal Collection (KAC). Strains of *Anabaena* (KAC 16), *Aphanizomenon* (KAC 15, KAC 69), and *Nodularia spumigena* (KAC 11, KAC 13, KAC 71) were grown in sterile Erlenmeyer flasks containing silica-free modified f/2 medium with a salinity of 7 PSU [35]. The cultures were maintained at 20 °C under irradiance of 104–108 μmol photons m^−2^ s^−1^ on a light/dark cycle of 16:8 h. After 31 days of growth, all six cyanobacterial cultures were filtered in steps through 80, 10, and 5 μm nylon nets to remove the culture medium and the remaining biomass was mixed into 0.2 μm filtered Baltic Sea water (polypropylene filter cartridge, Roki Techno). Chlorophyll-a was used as a proxy to determine cyanobacterial biomass from each strain culture. Aliquots of cultures extracted with 96% ethanol were measured using a 10-AU fluorometer (Turner Designs) [36]. Chlorophyll-a values in relation to microscope cell counts were used to determine the volume of each strain to be added to the sediment cores. The cultures had a range of 69–225 μg/L chlorophyll-a and were later confirmed in replicate cultures to consist of ~ 11,000 to ~ 108,000 cells per mL depending on the cultured strain (Supplementary Table 1). The cyanobacteria strains were mixed and consisted of *Nodularia spumigena* (59%), *Aphanizomenon* (32%) and *Anabaena* (9%), representative of a late summer bloom in the Western Gotland Sea [37]. The final cyanobacteria mix was kept in darkness at 12.5 °C for 7 days after which it was added to the cores. To determine the amount of cyanobacteria to add in each core, the mixed culture was homogenized and 2 mL transferred into pre-weighed microcentrifuge tubes (number of biological replicates (*n*) = 3) as well as controls containing 0.2 μm filtered Baltic Sea water (*n* = 3). The tubes were dried at 70 °C and re-weighed to determine the dry weight (dw). Before the incubation experiment, 1.4 to 2.4 dw g m^−2^ cyanobacteria were added to eight sediment cores (with the remaining six cores as controls). This corresponded to 0.5 to 1.0 dw g C m^−2^ using a 40% dw-carbon factor [38]. This concentration was within the lower range of previously published amounts of cyanobacteria biomass that reach the sediment [24, 39].

### Spring Sampling and Preparation of Diatom Biomass

Sediment cores (average sediment height of 22.4 cm) were collected for the spring experiment from the same coastal site on 16 April 2015. Twenty-one cores were sampled, three sliced in the field, water and sediment retained for chemical and biological analyses, and transported as described above.

Diatom strains were obtained from the Finnish Environment Institute SYKE. Strains of *Chaetoceros wighami* (CWTW C1), *Thalassiosira baltica*, *Skeletonema marinoi* (SMTV 1), *Melosira artica* (MATV 1), and *Diatoma tenuis* (DTTV B5) that are typical for the Baltic Sea spring bloom [40]. All strains were grown in modified f/2 medium with a salinity of 7 PSU [35]. The cultures were maintained at 16 °C under irradiance of 140 μmol photons m^−2^ s^−1^ on a light/dark cycle of 18:6 h. After 80 days of growth, the strains were mixed and the community composition determined by microscopy (~ 54,000 to ~ 179,000 cells per mL depending on the cultured strain) and contained *C. wighami* (11%), *T. baltica* (18%), *S. marinoi* (27%), *M. artica* (35%), and *D. tenuis* (9%) (Supplementary Table 1). The mixed community was then filtered through 1 μm nylon net to remove the medium (100 mL per time) and the net was sprayed with filtered seawater to collect the biomass in 50 mL centrifuge tubes. After all the culture had been collected, it was transferred into sterile petri dishes. Diatom biomass was then dried at 30 to 60 °C for ~ 3 h and the content (dry diatom biomass and sea salt) from all the petri dishes was combined. The salt content in grams was determined with a control consisting of 0.2 μm filtered sea water. After the sediment cores had been sampled, 75 mg diatoms plus salt mix was added to each of eight sediment cores (the remaining seven cores were controls). This corresponded to 0.2 dw g C m^−2^ (determined by CN analysis, PerkinElmer, Series II Analyzer 2400) and represented a ~ 1 day of decomposition of diatom biomass and was within the lower range of previously published sedimentation of diatoms [23, 41].

### Incubation of Sediment Cores

At the start of the experiments, three cores were sacrificed as zero time points and water plus sliced sediment were collected as described above. Sediment cores were incubated with addition of cyanobacterial biomass in darkness for 21 days at 12.5 °C (incubations conducted after the autumn sampling) or diatom biomass for 21 days at 11 to 13 °C (incubations conducted after the spring sampling). To ensure added biomass sank to the sediment surface, the cyanobacteria or diatom biomass was added 1 day before the incubation experiment was started without mixing. The water phase in all sediment cores was exposed to the air and was mixed using submersed sterile 15 mL tubes containing neodymium magnets attached to a monofilament line attached to the core lid. Neodymium magnets rotating outside the sediment cores were used to gently mix the water phase. Sediment cores were divided into treatments: i. water phase gently bubbled with air (flow rate 20 cm^−3^ s^−1^; *n* = 3 to 4; designated as “Bubbling”); ii. water phase gently bubbled with air and added cyanobacteria or diatom biomass (*n* = 4; designated as “Bubbling + Cyano/Diatoms”); iii. no bubbling, i.e., only air-water interface diffusion of oxygen (*n* = 3; designated as “No bubbling”); and iv. no bubbling with added cyanobacteria or diatom biomass (*n* = 4; designated as “No bubbling + Cyano/Diatoms”). An overview of the incubation design is available in Table 1. During incubation, 16 mL water was periodically collected from each core for water chemistry along with 50 mL for DNA (filtered and stored until DNA extraction as described earlier). At each sampling point, dissolved oxygen was measured in the water column and twice in the top 7 mm of sediment surface using an optical oxygen sensor in combination with a 50 μM resolution micromanipulator (FireStingO2; OXR50 oxygen sensor; Micromanipulator MU1, Pyroscience). At the end of the incubation experiment, water was collected and sediment sliced as described earlier. For the sediment sampled in spring, some cores were sliced after 9 days.

**Table 1:** Incubation setup and percentage of sediment organic matter (OM) in the sediment from both the autumn and spring experiments

Experiment	Bubbling/no bubbling + biomass	Day	OM % wt	*n*
Autumn	Sediment field		14.31 ± 0.38	3
Autumn	Sediment zero time point	0	13.64 ± 0.24	3
Autumn	No bubbling + cyano	21	13.19 ± 1.13	4
Autumn	No bubbling	21	13.58 ± 0.68	3
Autumn	Bubbling + cyano	21	13.12 ± 0.66	4
Autumn	Bubbling	21	13.60 ± 0.49	3
Spring	Sediment field		13.65 ± 0.14	3
Spring	Sediment zero time point	0	13.43 ± 0.74	3
Spring	No bubbling + diatoms	9	13.14–15.18	2
Spring	No bubbling + diatoms	21	13.99–23.90	2
Spring	No bubbling	9	13.33	1
Spring	No bubbling	21	13.56–14.31	2
Spring	Bubbling + diatoms	9	13.88–14.13	2
Spring	Bubbling + diatoms	21	13.26–13.85	2
Spring	Bubbling	9	13.41–13.82	2
Spring	Bubbling	21	13.85–13.97	2

The water phase in all sediment cores was exposed to air-water interface oxygen diffusion, mixed using magnets, and a portion of the cores were additionally aerated by bubbling air in the water. The OM measurements were conducted in the 0–1 cm sediment layer. Replicates indicate individual sediment cores (ranges are given for two or less replicates), SD = 1

### Chemistry Measurements

Chemistry data of the water phase and the top 1 cm sediment was measured in the field and at the end of the incubations and the water phase in the sediment cores was subsampled typically every fourth day. Directly after subsampling the sediment cores, all water samples were passed through a 0.7-μm glass fiber filter (GF/F filter, Whatman or 30-SF-07 (GMF) syringe filter, Chromacol). To measure the chemistry in the sediment pore-water, the sliced sediment samples were centrifuged at 2200*g* for 15 min and the collected supernatant was passed through a 0.7-μm filter. A DR 5000 Hach-Lange spectrophotometer was used to measure PO_4_^3−^ with the molybdenum blue method [42], NO_2_^−^ in combination with NO_3_^−^ with the naphthylethylenediamine method [42], ammonium (NH_4_^+^; with a Hach-Lange LCK 304 kit) and SO_4_^2−^ (LCK 353). The ferrozine method was used to measure total iron with a SmartSpec 3000 Bio-Rad spectrophotometer [43]. pH and redox potential were measured with a pHenomenal VWR pH electrode and Ag^0^/AgCl SI Analytics electrode (Mettler Toledo), respectively. To determine OM (% wt) of the sliced sediment, loss on ignition was conducted by igniting dried sediment at 550 °C for 4 h using a muffle furnace (OWF 1200, Carbolite) and the OM percentage calculated from the decrease in weight before and after ignition.

### DNA Extraction, Sequencing, and Bioinformatic Analysis

The frozen water phase filters and sediment samples were extracted for DNA using the PowerWater DNA Isolation Kit and PowerSoil DNA Isolation Kit (MO BIO Laboratories), respectively. DNA concentrations were determined with a NanoDrop 2000. All extracted samples were kept at − 80 °C until Illumina library preparation. A portion of the 16S rRNA gene was amplified using primers 341f and 805r [44] using a modified PCR program by [45] as previously described [46]. The Illumina library was sequenced at the Science for Life Laboratory, Stockholm using Illumina MiSeq pair-ends (2 × 301 bp). The UPARSE pipeline was used to quality filter and assembly the sequence data [47] and operational taxonomic units (OTUs; based on 97% threshold similarity) annotated against the SINA/SILVA NR99 version 119 (spring experiment) and 123 database (autumn experiment) [48]. The final data was analyzed using the software Explicet [49]. Shannon’s H alpha diversity index was calculated after OTU counts were sub-sampled to the lowest sample size and 100 × bootstrap (autumn experiment: 4094 and the spring experiment: 40044 counts). Maximum likelihood phylogenetic trees were constructed in MEGA 7 after aligning sequences using MUSCLE (using 8 iterations). The trees were unrooted, bootstrapped 100 times, and based on the Tamura-Nei model with nucleotide substitution [50]. Principal component analysis of OTU groups was constructed with the use of the software past 3.10 [51]. Reference sequences for close relatives were retrieved from the NCBI taxonomy database. A list of the number of raw read pairs, after merging, quality trimming, and the amount of clustered OTUs is available in Supplementary Table 2. The 16S rRNA gene sequences for the autumn and spring experiments are available on the NCBI database with the BioProject accession number PRJNA323408.

## Results

Dissolved O_2_ concentrations were > 7 mg/L in the sediment surface regardless if biomass had been added or not and did not change in penetration depth (~ 0.5 cm) and concentration throughout the incubations. This was likely due to the stated aim of adding small amounts of biomass to the sediment cores. O_2_ in the water phase within the sediment cores was 8 to 10 mg/L and the pH remained at ~ 7.5 throughout both the autumn (cyanobacteria) and spring (diatom) incubation experiments. The redox potential in the sediment surface was in the range of 100 to 120 mV for the autumn experiment and ~ 150 mV for the spring experiment. A complete list of oxygen values in the water phase is available in Supplementary Table 3 while a full list of pH, redox values, and chemical measurements is available in Supplementary Table 4.

Principal component analysis of the relative abundances of OTUs grouped into the lowest available taxonomical level showed that two microbial communities were distinct, likely as they were sampled in the autumn and spring for the respective experiments (Supplementary Fig. 1). The spring community had a higher alpha diversity in the field (Shannon’s H index 11.11 ± 0.04) compared to autumn (9.53 ± 0.16) (*n* = 3 for both; one-way ANOVA with a post hoc multiple comparison Tukey; *p* < 0.05; Supplementary Table 5).

### Autumn Experiment: Water and Sediment Chemistry

**Fig. 1:**
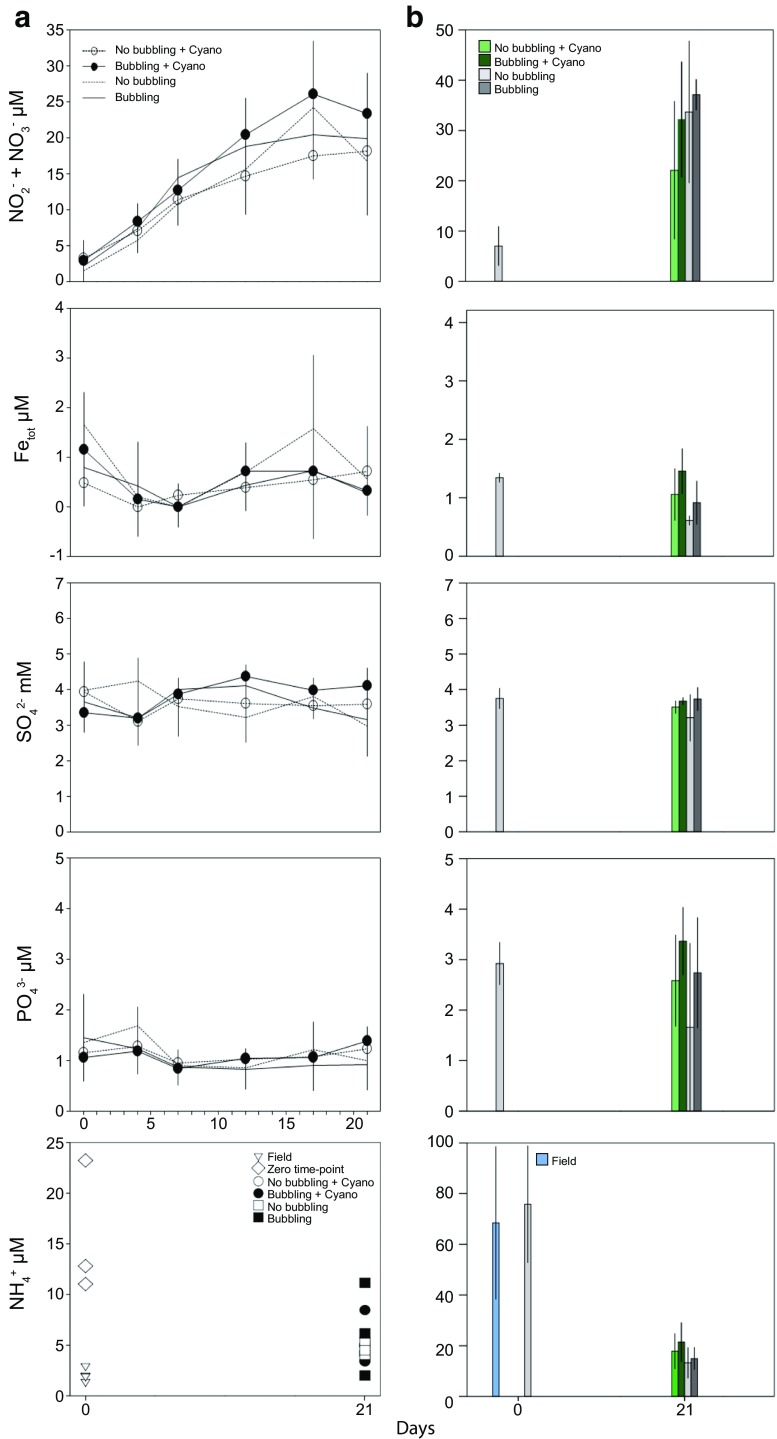
Chemistry data from the autumn experiment. **a** shows chemistry in the water phase. Symbols and lines denote: white circles, “No bubbling + Cyano”; black circles, “Bubbling + Cyano”; dotted lines, “No bubbling” plus black lines, “Bubbling.” Additional symbols for NH_4_^+^ measurements denote: open triangles, field; diamonds, zero time-point cores; white squares, “No bubbling”; and black squares, “Bubbling.” **b** shows sediment pore water chemistry data from the top 1 cm sediment. Colors on bar charts denote: blue, field; light gray (day 0 of incubation), zero time-points; light green, “No bubbling + Cyano.” Dark green, “Bubbling + Cyano”; Light gray, “No bubbling” and dark gray, “Bubbling.” Day 0 time points are an average of three biological replicates. All values with biomass are averages of four and without biomass three replicates, SD = 1 for all values

At the time of sampling in autumn 2014, the water had a salinity of 6.5 ‰, temperature 12.6 °C, pH 7.88, and a dissolved O_2_ concentration of 10.0 mg/L. The NO_2_^−^ + NO_3_^−^ concentration in the water phase was below 3 μM at the start of the autumn experiment but increased to 15 to 30 μM after 21 days (one-way ANOVA, *F* = 106.32, *p* < 0.01; Fig. 1). However, there was no statistically significant difference between the end values for all of the treatments. At the end of the incubations, the NO_2_^−^ + NO_3_^−^ concentrations in the water phase were (averages ± 1 SD for all values): cores not bubbled (“No bubbling” and “No bubbling + Cyano”), 17.6 ± 1.52 μM (*n* = 7) and bubbled with air (“Bubbling” and “Bubbling + Cyano”), 21.9 ± 2.58 μM (*n* = 7). NH_4_^+^ in the water phase showed no statistical significance upon biomass addition, instead the concentration significantly decreased from the zero time-points (15.7 ± 6.6 μM, *n* = 3) to all end points (5.13 ± 2.29 μM, *n* = 14, *p* < 0.05; Fig. 1). In the top 1 cm sediment, the NO_2_^−^ + NO_3_^−^ concentration was initially 7.05 ± 3.91 μM (*n* = 3) and increased to 20 to 40 μM at the end of the incubation (*F* = 5.24, *p* < 0.05; Fig. 1). At the end of the incubations, NO_2_^−^ + NO_3_^−^ concentrations for the top 1 cm sediment were: added biomass (“Bubbling + Cyano” and “No bubbling + Cyano”) 27.1 ± 6.3 (*n* = 8) and no biomass (“Bubbling” and “No bubbling”) 35.4 ± 4.3 (*n* = 6) (no statistical significance). NH_4_^+^ in the sediment pore-water showed no statistical significance with biomass addition. Instead a significant decrease was observed when comparing field data (84.08 ± 19.68 μM, *n* = 3) and zero-time points (62.64 ± 23.61 μM, *n* = 3) to all end points (16.96 ± 6.45 μM, *n* = 13, *p* < 0.05; Fig. 1). Organic matter (% wt) in the sediment correlated positively with NO_2_^−^ + NO_3_^−^ (Pearson correlation: *p* < 0.045, *r* = 0.453). Total iron concentrations were below 2 μM in the water phase and the top 1 cm sediment layer throughout the incubation period while sulfate concentrations showed no trend with addition of cyanobacteria biomass (Fig. 1). In the water phase, the PO_4_^3−^ concentrations were < 3 μM while in the sediment surface, the concentrations were < 4 μM throughout the experiment (Fig. 1). After 21 days of incubation sediment OM in the “No bubbling + Cyano” and “Bubbling + Cyano” treatments was stable in the sediment surface with values of 13.64 ± 0.24% (*n* = 3) at the start of the incubation and 13.19 ± 1.13 and 13.12 ± 0.66 at the end, respectively (both *n* = 4; Table 1). Sediment cores without addition of cyanobacteria also remained stable at 13.58 ± 0.68 and 13.60 ± 0.49 in the “No bubbling” and “Bubbling” treatments, respectively.

### Autumn Experiment: Microbial Community Changes in Response to Cyanobacteria Biomass

**Fig. 2:**
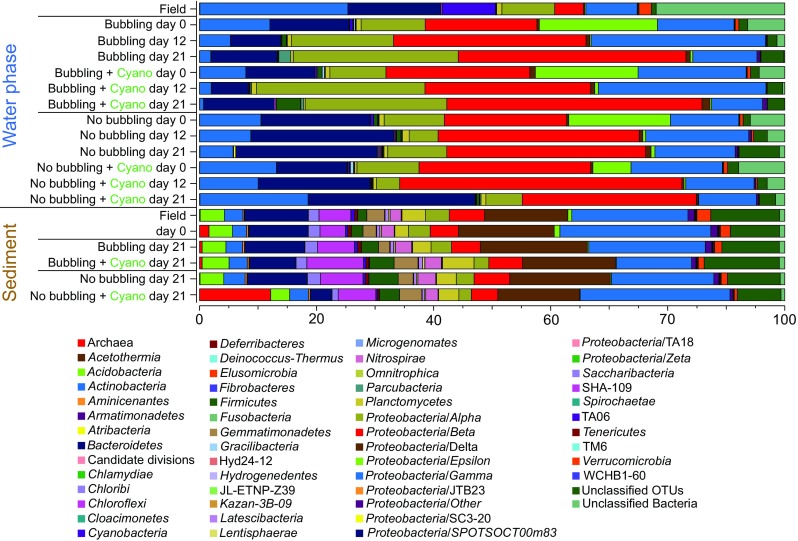
Stacked bars from the autumn experiment of the relative abundance of microbial communities in the water phase and the sediment surface. Taxonomical level is on phylum expect proteobacteria which has been divided into classes. The relative abundance was calculated as the average of the biological replicates. The replicates for the water samples are: field (*n* = 4); Bubbling (*n =* 3 except day 21 where *n* = 2); “Bubbling + Cyano” day 0 (*n* = 4); “Bubbling + Cyano” day 12 (*n* = 2); “Bubbling + Cyano” day 21 (*n* = 3); “No bubbling” (*n* = 3); and “No bubbling + Cyano” (*n* = 4 except day 21 where *n* = 3). The replicates for the sediment samples are: field (*n* = 4); zero time-point (*n* = 3); “Bubbling” (*n* = 3); “Bubbling + Cyano” (*n* = 4); “No bubbling” (*n* = 3); and “No bubbling + Cyano” (*n* = 4)

**Fig. 3:**
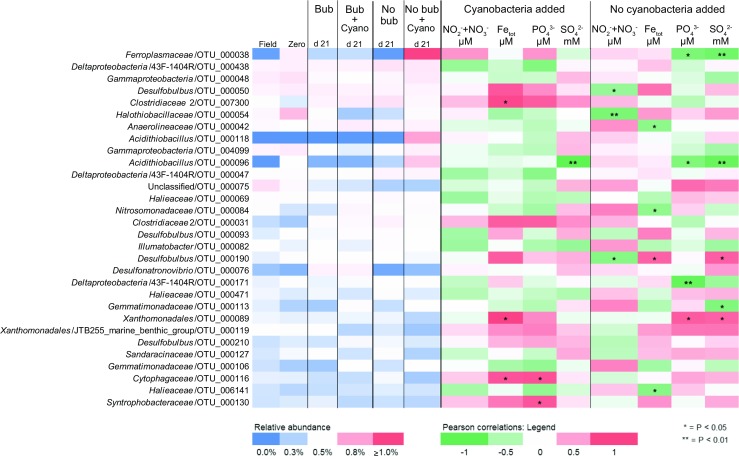
The top 30 most abundant OTUs in the 0–1 cm sediment surface from the autumn experiment. Relative abundance is shown on the left side (bubbling abbreviated as bub) while Pearson correlations on the right side were used to find trends among the OTUs in relation to chemistry fluxes. Samples were divided into two groups, cyanobacteria added (*n* = 12–15) and not added (*n* = 10–13)

**Fig. 4:**
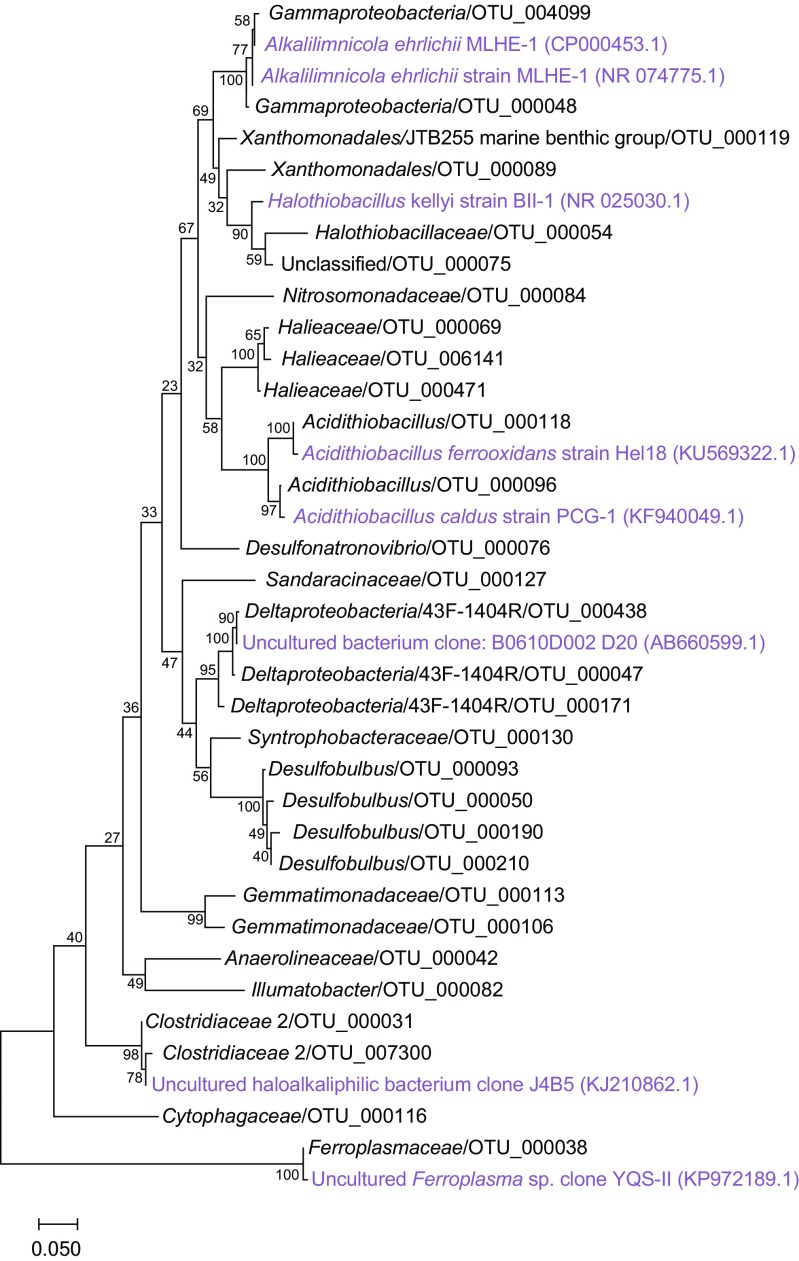
Phylogenetic maximum likelihood tree of the top 30 abundant OTUs from the autumn experiment (100 × bootstrap). The unrooted tree with the highest log likelihood (− 4149.1679) is shown. Reference sequences of close relatives (light purple text) were found by searching unclassified OTUs against the NCBI taxonomy database

The microbial communities dominating the water phase during autumn aligned with the *Actinobacteria*, *Bacteroidetes*, *Alpha*-, *Beta-*, and *Gammaproteobacteria*. Dominant microbial communities in the sediment surface were *Bacteroidetes*, *Chloroflexi*, *Delta-*, and *Gammaproteobacteria*, and unclassified OTUs (Fig. 2). A full list of annotated OTUs from the autumn experiment can be found in Supplementary Data 1. The sediment cores in the autumn experiment had a statistically significant increase in *Firmicutes* from 0.9 to 4.1% after 21 days in the water phase in the “Bubbling + Cyano” cores (post hoc Tukey test, *p* < 0.05, *n* = 3; Fig. 2). The sediment surface of the “No bubbling + Cyano” treatment showed an increase in archaea in two of four sediment cores from 1.54 ± 2.59% at day 0 (*n* = 3) to 11.67 ± 15.23% at day 21 (*n* = 4; Fig. 2). One-way ANOVA with a post hoc multiple *comparison* Tukey test showed a statistically significant difference in the relative proportion of archaea in the “No bubbling + Cyano” sediment after 21 days of incubation compared to “No bubbling” sediment without cyanobacteria (*p* < 0.05) as well as the sediment sliced in the field (*p* < 0.05). This was also observed in the principle component analysis (Supplementary Fig. 1) and Shannon’s H alpha diversity index (One-way ANOVA with a post hoc multiple *comparison* Tukey, *p* < 0.05; Supplementary Table 5). The increase of archaea belonged to a single OTU aligning with the class *Thermoplasmatales* and family *Ferroplasmaceae* (Fig. 3 and Supplementary Data 1). Pearson correlations between chemistry and taxonomic lineages in the sediment surface showed that archaea only significantly negatively correlated with OM (*p* < 0.047, *r* = −450). Additional Pearson correlations of the top abundant 30 OTUs and the chemistry measurements in the 0 to 1 cm sediment surface were conducted in two groups: sediment cores with added cyanobacteria (*n* = 12 to 15) and sediment cores without added cyanobacterial biomass (*n* = 10 to 13; Fig. 3 and Supplementary Table 6). Of the top 30 abundant OTUs, two were annotated to the genus *Acidithiobacillus* from the *Acidithiobacillia* class [previously Gammaproteobacteria; [52]] with one appearing in all cores in low abundance (OTU 96) and the other OTU occurring in higher abundance in only one of the “No bubbling + Cyano” sediment cores (OTU 118, 23.92%; Fig. 3 and Supplementary Data 1). The *Acidithiobacillus* OTU that appeared in all sediment treatments negatively correlated to SO_4_^2−^ but not depending on addition of cyanobacterial biomass (*p* < 0.01, added cyanobacteria *r* = − 0.87; no added cyanobacteria *r* = − 0.83). These two *Acidithiobacillus* OTUs were most closely related to the species *A. ferrooxidans* and *A. caldus* based on the phylogenetic tree containing reference species from the NCBI taxonomy database (Fig. 4). In the same phylogenetic tree, two unclassified OTUs belonging to *Gammaproteobacteria* were related to the halophilic bacteria species *Alkalilimnicola ehrlichii* and one OTU with the genus *Halothiobacillus*. Interestingly, both acidophilic and halophilic bacteria were observed in the sediment surface but neither were significantly affected by addition of cyanobacteria biomass. One OTU annotated to the family *Cytophagaceae* belonging to the *Bacteroidetes* was found to be positively correlated to total iron and PO_4_^3−^ in treatments with cyanobacterial addition (*p* < 0.05, *r* = 0.72 and 0.73, respectively). This could possibly be due to precipitation of ferric-phosphate indicating favorable aerobic conditions in the sediment.

### Spring Experiment: Water and Sediment Chemistry

**Fig. 5:**
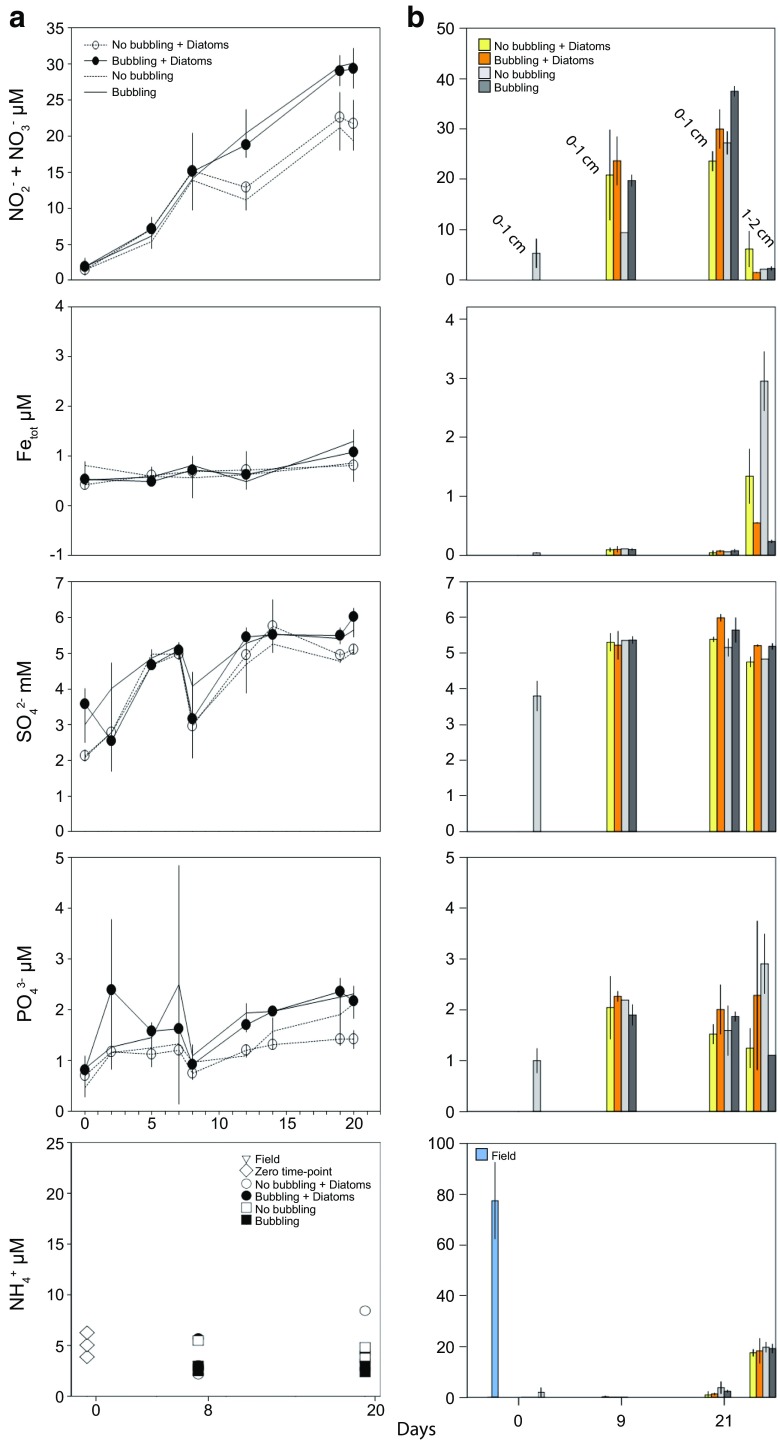
Chemistry data from the spring experiment. **a** shows chemistry in the water phase. Symbols and lines denote: white circles, “No bubbling + Diatoms”; black circles, “Bubbling + Diatoms”; dotted lines, “No bubbling” plus black lines, “Bubbling.” Additional symbols for NH_4_^+^ measurements denote: open triangles, field; diamonds, zero time-point cores; white squares, “No bubbling”; and black squares, “Bubbling.” **b** shows sediment pore water chemistry data from the 0–1 and 1–2 cm sediment slices. Colors on bar charts denote: blue, field; light gray (day 0 of incubation), zero time-points; light green, “No bubbling + Diatoms.” Dark green, “Bubbling + Diatoms”; Light gray, “No bubbling” and dark gray, “Bubbling.” Day 0 time points are an average of three biological replicates. Values with biomass are averages of four cores, except day 9 and day 21 (*n* = 2). Cores without biomass consisted of “No bubbling” (*n* = 3) and “Bubbling” (*n* = 4), except on day 9 (all *n* = 2, except “No bubbling” *n* = 1), and day 21 (all *n* = 2), SD = 1 for all values

In spring 2015, the water in the field had a salinity of 6.2 ‰, temperature 6.2 °C, pH 8.35, and a dissolved O_2_ concentration of 13.7 mg/L. The spring experiment had similar NO_2_^−^ + NO_3_^−^ concentrations in the water phase as the autumn experiment and was below 3 μM at the start of the experiment and increased to 15 to 30 μM after 20 days (*F* = 287.60, *p* < 0.01; Fig. 5). Cores gently bubbled with air had a higher increase in NO_2_^−^ + NO_3_^−^ compared to the cores without bubbling (*F* = 33.22, *p* < 0.05), but addition of diatom biomass showed no significant effect on the NO_2_^−^ + NO_3_^−^ concentrations. NO_2_^−^ + NO_3_^−^ concentrations at the end of the experiment in the water phase were on average: not bubbled (“No bubbling” and “No bubbling + Diatoms”) 20.5 ± 1.13 μM (*n* = 4) and bubbled (“Bubbling” and “Bubbling + Diatoms”) 29.8 ± 1.2 μM (*n* = 4). NH_4_^+^ in the water phase showed no statistical significance with biomass addition or when comparing the zero time-points (5.06 ± 1.19 μM, *n* = 3) to all end points (4.32 ± 1.83 μM; Fig. 5). The initial NO_2_^−^ + NO_3_^−^ concentration in the top 1 cm sediment was 5.34 ± 2.93 μM (*n* = 3) that increased to 20-40 μM at the end of the incubation, (*F* = 46.181, *p* < 0.01; Fig. 5). NO_2_^−^ + NO_3_^−^ concentrations in the sediment at the end of the experiment were: added diatoms (“No bubbling + Diatoms” and “Bubbling + Diatoms”) 26.8 ± 1.47 (*n* = 4) and no diatoms (“No bubbling” and “Bubbling”) 32.4 ± 0.84 (*n* = 4) (no statistical significance). In the 1 to 2 cm sediment slices, the NO_2_^−^ + NO_3_^−^ concentrations were significantly lower than in the 0 to 1 cm fraction (*F* = 8.75, *p* < 0.05). NH_4_^+^ in the sediment pore-water showed no statistical significance upon biomass addition. Instead a significant decrease was observed between the field data (74.65 ± 14.62 μM, *n* = 3) to all end point 0–1 cm and 1–2 cm sediment slices (10.41 ± 8.77 μM, *n* = 16, *p* < 0.01; Fig. 5). In addition, there was significantly lower NH_4_^+^ in the 0–1 cm sediment slices (*n* = 8) when compared to the 1–2 cm slices (*n* = 8) (2.13 ± 1.61 compared to 18.69 ± 2.38, *F* = 265.5, *p* < 0.01).

Total iron concentrations were < 2 μM throughout the incubation period in the water phase and top 1 cm sediment layer (Fig. 5). In contrast, the total iron concentrations in the 1 to 2 cm sediment slices were significantly higher compared to the top 1 cm layer (*F* = 144.60, *p* < 0.01). There was also a difference depending if diatom biomass was added to the sediment surface: “No bubbling + Diatoms” 20.11 ± 3.48 (*n* = 2); “No bubbling,” 44.28 ± 3.78 (*n* = 2); “Bubbling + Diatoms,” 8.24 ± 0.08 (*n* = 2); and “Bubbling,” 3.53 ± 0.23 (*n* = 2). The “No bubbling + Diatoms” was significantly different with “No bubbling” (one-way ANOVA Tukey post hoc test, *p* < 0.05). There was a continuous increase in SO_4_^2−^ in the water (*F* = 93.74, *p* < 0.01; Fig. 5) and sediment phases (*F* = 45.48, *p* < 0.01; Fig. 5). However, no significant change could be observed with regard to biomass addition. PO_4_^3−^ concentrations were < 3 μM in the water phase and < 4 μM in the sediment surface throughout the experiment (Fig. 5). The 1 to 2 cm sediment slices also had PO_4_^3−^ concentrations < 4 μM and showed a no statistical significance depending on biomass addition: “No bubbling + Diatoms,” 1.25 ± 0.39 (*n* = 2); “No bubbling,” 2.91 ± 0.59 (*n* = 2); “Bubbling + Diatoms,” 2.28 ± 1.47 (*n* = 2); and “Bubbling,” 1.11 ± 0.00 (*n* = 2). The percentage of sediment OM had no statistical significant differences with 13.43 ± 0.74% (*n* = 3) at the start of the incubation and 18.94 ± 7.01 and 13.56 ± 0.42 after 21 days incubation in the “No bubbling + Diatoms” and “Bubbling + Diatoms” treatments, respectively (both *n* = 2; Table 1). In the treatments without addition of biomass, the percentage of OM was also stable with 13.94 ± 0.53% and 13.94 ± 0.53% in “No bubbling” and “Bubbling,” respectively (both *n* = 2).

### Spring Experiment: Microbial Community Changes in Response to Diatom Biomass

**Fig. 6:**
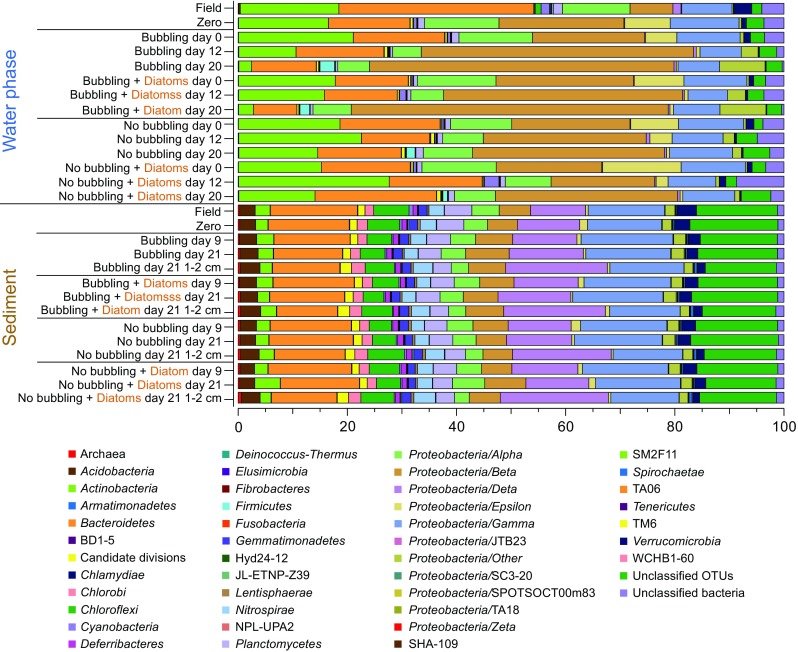
Stacked bars from the spring experiment of the relative abundance of microbial communities in the water phase and the sediment surface. Taxonomical level is on phylum expect proteobacteria which has been divided into classes. The relative abundance was calculated as the average of the biological replicates. The replicates for the water samples are: field (*n* = 3); zero time point (*n* = 3); day 0 “Bubbling” (*n* = 4), “No bubbling + Diatoms” (*n* = 4), “Bubbling + Diatoms” (*n* = 3), “No bubbling” (*n* = 3); day 12 (*n* = 2); day 20 (*n* = 2). The replicates for the sediment samples are: field (*n* = 3); zero time-point (*n* = 3); day 9 (*n* = 2 except “No bubbling” where *n* = 1); and day 21 (*n* = 2)

**Fig. 7:**
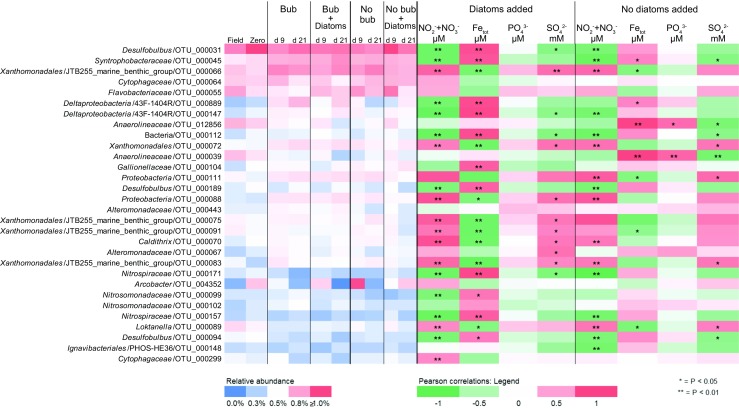
The top 30 most abundant OTUs in the 0–1 cm sediment surface from the spring experiment. Relative abundance is shown on the left side (bubbling abbreviated as bub) while Pearson correlations on the right side were used to find trends among the OTUs in relation to chemistry fluxes. Samples were divided into two groups, diatoms added (*n* = 8) and not added (*n* = 7)

**Fig. 8:**
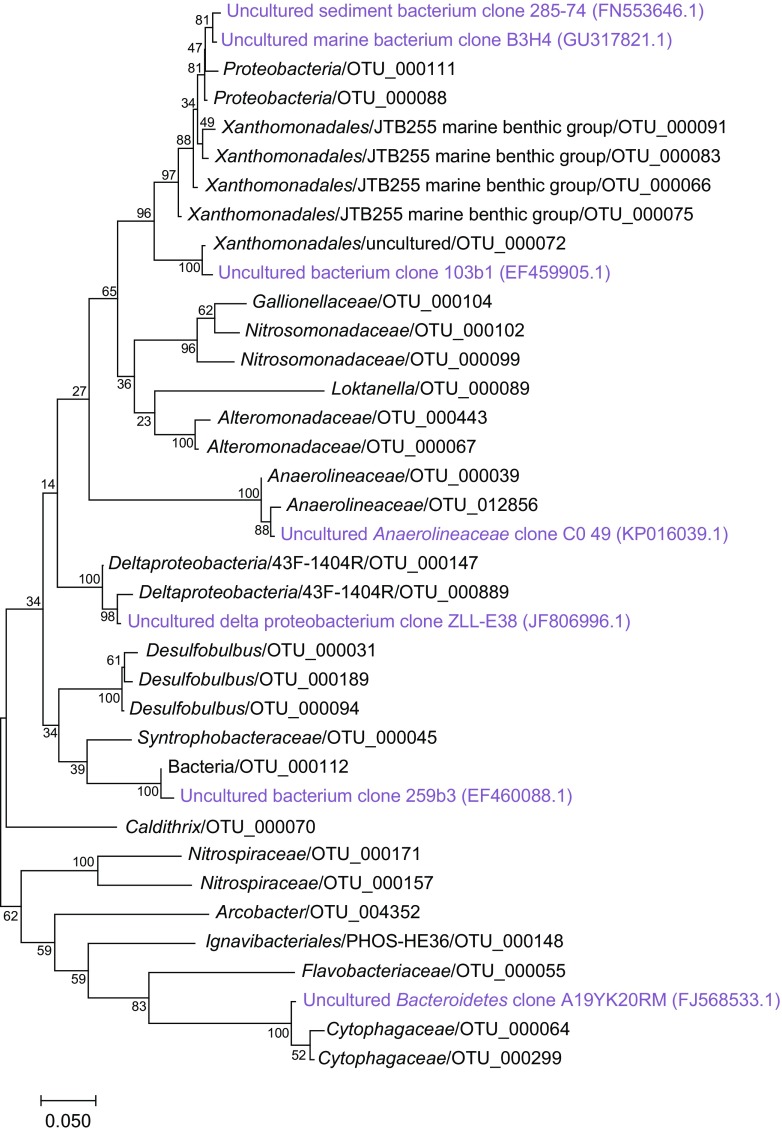
Phylogenetic maximum likelihood tree of the top 30 abundant OTUs from the spring experiment (100 × bootstrap). The unrooted tree with the highest log likelihood (− 4840.1258) is shown. Reference sequences of close relatives (light purple text) were found by searching unclassified OTUs against the NCBI taxonomy database

Similar to the autumn experiment, microbial communities dominating the water phase aligned within the *Actinobacteria*, *Bacteroidetes*, *Alpha*-, *Beta-*, and *Gammaproteobacteria*. Dominant microbial communities in the sediment surface were *Bacteroidetes*, *Chloroflexi*, *Delta-*, and *Gammaproteobacteria*, and unclassified OTUs (Fig. 6). A full list of annotated OTUs from the spring experiment is in Supplementary Data 2. At the phylum and *Proteobacteria* class level in the spring experiment, no obvious changes in relative abundance were seen in the water or sediment after diatom biomass had been added (Fig. 6), neither was any difference observed in sediment microbial diversity based on Shannon’s H index. In the sediment surface after 21 days (“No bubbling,” “No bubbling + Diatoms,” “Bubbling” and “Bubbling + Diatoms”; *n* = 2 for each treatment), the classes *Alpha*- (~ 4%), *Beta*- (~ 6%), *Delta*- (~ 13%), and *Gammaproteobacteria* (~ 15%) were among the most abundant *Proteobacteria* phyla. Other abundant classes were *Bacteroidetes* (~ 14%), *Chloroflexi* (~ 5%), and *Planctomycetes* (~ 4%). In more detail, population increases in the *Betaproteobacteria* were in the order *Burkholderiales* in the “No bubbling + Diatoms” treatment as a result of a higher relative abundance of the genus BAL58 marine group aligning to the family *Comamonadaceae* (Supplementary Data 2). The 30 most abundant OTUs in the samples revealed that the genus *Desulfobulbus* belonging to *Deltaproteobacteria* was the most copious (Fig. 7), especially in the depth range 1–2 cm (> 1% relative abundance; Supplementary Data 2). Also belonging to the *Deltaproteobacteria*, one of the most abundant OTUs (~ 1% relative abundance) aligned within the family *Syntrophobacteraceae*. Sediment cores that had diatoms added but not bubbled with air had an increase in one OTU (of the top 30) after 9 days incubation belonging to the genus *Flavobacteriaceae* (Fig. 7). The relative abundance of this OTU was higher in cores bubbled with air and with added diatoms (~ 0.65%) when compared to cores only bubbled with air (~ 0.45%; *n* = 2 for each treatment). Another OTU aligning to the genus *Arcobacter* decreased when diatoms were added in conjunction with bubbling of air while it increased after 21 days when diatoms were added without the bubbling treatment (*n* = 2 for each treatment). Pearson correlations of the top 30 OTUs and the chemistry measurements in the 0 to 1 cm sediment surface were conducted in two groups: sediment cores with added diatoms (*n* = 8) and sediment cores without added diatoms (*n* = 7; Fig. 7 and Supplementary Table 6). One unclassified bacteria significantly correlated positively with NO_2_^−^ + NO_3_^−^ independent of diatom addition (*p* < 0.01, *r* = − 0.8) and negatively with total iron (*p* < 0.01, *r* = 0.87) in cores amended with diatom biomass. The phylogenetic tree showed that this and other uncultured bacteria in the top abundant OTUs remained unknown after comparison against the NCBI taxonomy database (Fig. 8). A few OTUs annotated to the order *Xanthomonadales* belonging to the class *Gammaproteobacteria* were positively correlated to NO_2_^−^ + NO_3_^−^ and SO_4_^2−^ (*p* < 0.01, *r* = ~ 0.7 and *p* < 0.01, *r* = ~ 0.6, respectively) while one OTU was negatively correlated to total iron (*p* < 0.01, *r* = ~ − 0.8). One OTU annotated to the genus *Nitrosomonadaceae* belonging to the *Betaproteobacteria* was found to be negatively correlated to NO_2_^−^ + NO_3_^−^ (*p* < 0.01, *r* = − 0.7).

## Discussion

In the autumn experiment, sediment cores not bubbled with air and amended with cyanobacterial biomass (“No bubbling + Cyano”) had a significant increase in relative abundance of *Ferroplasmaceae* in the top 1 cm sediment (Fig. 3 and Supplementary Data 1). The archaea family *Ferroplasmaceae* consists of acidophilic aerobic or facultative anaerobic species with an optimal pH range of 0.8–1.8 and with a metabolic capability to oxidize ferrous iron and organic carbon [53]. However, it was surprising that a population most similar to a family of obligate extreme acidophiles was present in the field samples and subsequently favored during the incubation (Fig. 3). One explanation for this could be due to cross-contamination between samples in the 16S rRNA gene amplicon library. However, the dual-index approach used to construct the library reduces the error rate and chance of cross-contamination [54, 55]. Additionally, cross-contamination was considered unlikely as *Ferroplasmaceae* were observed in higher abundance in two of four sediment cores in the “No bubbling + Cyano” treatment and OTUs aligning with *Acidithiobacillia* spp. appeared in all four cores of the same treatment that was consistent with the presence of low pH adapted populations (Supplementary Data 1). A potential explanation could be that *Ferroplasmaceae* spp. were present in microniches, as previously reported in acidic microenvironments derived from sulfide oxidation [56]. That the potential for sulfide oxidation existed in the sediment in both the autumn and spring samples was indicated by sulfide oxidizing bacteria such as *Desulfobulbus* spp., *Sulfurovum* spp., and *Sulfurimonas* spp. in the 16S rRNA gene dataset (Supplementary Data 1). *Sulfurimonas* is known to oxidize sulfide in the oxic-anoxic interface in the sediment [57] and considering that oxygen penetration reached ~ 0.5 cm, the underlying anoxic layer was also sampled. *Sulfurimonas* is found in Baltic Sea anoxic waters [58] plus sediment [57] and has also been found in Baltic Sea surface water during December likely due to upwelling of the sediment and bottom waters [59]. Since both acidophilic (e.g., *Acidithiobacillia* spp.) and halophilic (*Alkalilimnicola ehrlichii* and *Halothiobacillus* sp.) bacteria were found in the most abundant taxa during autumn (Fig. 4), it was possible that microenvironments existed in the sediment that selected for populations with these particular characteristics. The occurrence of both acidophilic and halophilic bacteria in in situ oxic sediment has been observed previously at the same sampling site [34]. Considering that 1 cm sediment slices were analyzed, this functional diversity of different microbes likely derives from complex redox processes using different electron acceptors at a range of oxidation-reduction potentials as well as anoxic microniches. In addition, these findings indicated that just small alterations in oxygen availability (i.e., air-water interface diffusion compared bubbling the water phase with air) were sufficient to cause this change. Development of anoxic microniches in sediments have previously been observed after addition of diatomic biomass [60] and such anoxic microenvironments would likely be undetectable by the oxygen microsensor due to methodological difficulties to locate these zones. Therefore, it is suggested that the development of anoxic microniches in the sediment surface are initial processes after cyanobacteria biomass has settled on the sediment surface.

Addition of diatom biomass during the spring experiment resulted in a trend that sediment NO_2_^−^ + NO_3_^−^ concentrations were lower than the control cores. Similar findings were observed in the autumn experiment, but with a high variation. This could potentially have been due to nitrate reduction (decreasing the nitrification rate) upon addition of phytoplankton biomass [16]. However, isotope analysis indicated that addition of phytoplankton biomass actually suppressed denitrification [61]. In addition, Hansen and Blackburn [62] observed a temporary increase in denitrification after phytoplankton biomass addition which reverted after the OM had been degraded. These results indicated that early hypoxia development due to degradation of phytoplankton OM affected the nitrogen cycle in the sediment surface and further suggested that the differences observed in microbial composition can be explained by an increased microbial activity rather than wholesale changes in community structure [31]. For example, OTUs belonging to the genus *Desulfobulbus* were some of the most abundant in the dataset (Figs. 3 and 7). This genus is capable of sulfate reduction and sulfide oxidation utilizing NO_3_^−^ as a terminal electron acceptor [63, 64]. However, these changes in microbial composition were not surprising as the in situ microbial community in the sediment surface would have been adapted to sporadically degrading sinking OM derived from the water column. This microbial trait is important because degradation of OM lowers the availability of oxygen and initiates the development of “dead zones.”

Changes in chemistry in deeper sediment layers due to phytoplankton biomass addition to the sediment surface have been reported [16] and after diatom biomass addition in this study Fe concentration was lower and NO_2_^−^ + NO_3_^−^ higher in the 1-2 cm layer (Fig. 5). Iron reduction, typical in hypoxic and anoxic environments, has previously been observed to be stimulated after dried cyanobacterial biomass was added to sediment cores [65]. As these changes occurred, despite no observed decrease in the oxygen concentration, they could be key initial processes in the sediment that occur during the early stages of hypoxia development as a result of phytoplankton biomass sinking to the sediment.

Chemistry measurements indicated that the sediment at the field site contained a large amount of nitrogen compounds that were released as NO_2_^−^ + NO_3_^−^ into the water phase in all treatments. This was likely diffusion of stored nitrogen compounds from nearby agricultural lands plus active nitrification. This increase in NO_2_^−^ + NO_3_^−^ was also observed in a previous experiment with a similar setup and sediment sampled from the same site during 2013 [34]. That the sediment was initially loaded with nitrogen compounds was indicated by high concentrations of NH_4_^+^ in the field sediments (Figs. 1 and 5). NH_4_^+^ concentrations were also lower in the 0–1 cm sediment layer compared to the 1–2 cm layer, suggesting active NH_4_^+^ removal in the top 1 cm sediment surface (Fig. 5). Furthermore, OTUs aligning with nitrifying bacteria, e.g., *Nitrospira* spp. and *Nitrospina* spp. [66] were present in the sediment for both experiments (Supplementary Data 1 & 2). However, a mass balance of nitrogen compounds suggested that diffusion of the initial field NO_2_^−^ + NO_3_^−^ stored in the sediment combined with nitrification of NH_4_^+^ (Supplementary Data 7) provided 16 to 65% of the increase of NO_2_^−^ + NO_3_^−^ in the water phase for the autumn experiment and only 8 to 16% for the spring experiment. A possible explanation for the shortfall is the contribution by microbial anaerobic dissimilatory nitrate reduction in the deeper sediment producing NH_4_^+^ [67, 68]. This could be followed by NH_4_^+^ diffusion and nitrification in the oxic ~ 0.5 cm sediment surface and water column. Another process is N_2_-fixation that can contribute to the overall nitrogen budget in the system [69]. These findings highlight the importance of nitrogen cycling in benthic systems and it is suggested that cycling and conversion of NO_2_^−^_,_ NO_3_^−^, and NH_4_^+^ was driven by microbial processes.

## Conclusions

In this study, it was identified how the spring and autumn sediment microbial communities altered after addition of phytoplankton typical for the spring and summer bloom, respectively. This knowledge will help to elucidate early microbial community changes in the sediment before oxygen becomes scarce. During eutrophication events, the observed chemical and microbial changes could be key initial processes in the sediment during early hypoxia development. Interestingly, enforced aeration of the bottom water in conjunction with cyanobacteria addition did not cause these changes and consequently, oxygenation of the bottom water could potentially keep the microbial community structure in the sediment surface more stable during eutrophication events. In addition, the important role of nitrogen cycling was highlighted by increasing concentrations of NO_2_^−^ + NO_3_^−^ in the bottom water that occurred with or without phytoplankton biomass addition. This increase could not be explained by diffusion or nitrification of the measured NH_4_^+^. It is possible that microbial assimilation and cycling of nitrogen compounds increased the available nitrogen budget to account for this increase of NO_2_^−^ + NO_3_^−^ in the bottom water.

## Electronic Supplementary Material

ESM 1 (DOCX 220 kb)

Supplementary Data 1 The table shows the results as relative abundance from the annotation of clustered OTUs from the autumn cyanobacteria experiment. A separate Excel file has been uploaded. (XLSX 2478 kb)

Supplementary Data 2 The table shows the results as relative abundance from the annotation of clustered OTUs from the spring diatom experiment. A separate Excel file has been uploaded. (XLSX 4804 kb)
