# Supplementary material for: Spring and Late Summer Phytoplankton Biomass Impact on the Coastal Sediment Microbial Community Structure
Source: Microb Ecol. 2018 Jul 17;77(2):288–303. doi: 10.1007/s00248-018-1229-6 (PMC6394492; doi:10.1007/s00248-018-1229-6)
Supplement: Supplementary file 1 — (DOCX 220 kb) [file 248_2018_1229_MOESM1_ESM.docx]

Supplementary Material

Spring and late summer phytoplankton biomass impact on the coastal sediment microbial community structure

Elias Broman*, Lingni Li, Jimmy Fridlund, Fredrik Svensson, Catherine Legrand, Mark Dopson

*** Correspondence:** Elias Broman: elias.broman@lnu.se

**Supplementary Data 1.** The table shows the results as relative abundance from the annotation of clustered OTUs from the autumn cyanobacteria experiment. A separate Excel file has been uploaded.

**Supplementary Data 2.** The table shows the results as relative abundance from the annotation of clustered OTUs from the spring diatom experiment. A separate Excel file has been uploaded.

**Supplementary Table 1.** The experiment used chlorophyll-a (µg/L) to determine the volume to mix to form community composition of the autumn cyanobacterial experiment (Table S1a).

**Table S1a** Autumn cyanobacteria experiment conducted during 2014 October-November

Chlorophyll-a dilution series in relation to cyanobacteria cell counts were conducted during October and November 2016 using replicate cultures. Cyanobacterial samples for cell counts were stored in approx. 1% vol/vol Lugol prepared according to (Willén 1962). Chlorophyll-a values and cell counts were used to infer the slope (*k*) and calculate cell counts (Table S1b).

**Reference**

Willén T (1962). Studies on the Phytoplankton of Some Lakes Connected with or Recently Isolated from the Baltic. *Oikos* **13:** 169-199.

**Table S1b** The chlorophyll-a cyanobacteria cell relationship (2016 October-November) was used to determine cell counts from each cultured and final diluted/concentrated strains (using a 5 µm nylon net) of the autumn cyanobacteria experiment (2014) conducted during 2014 October-November. Based on cell counts this gave a final community composition of 59% *Nodularia* spp., 32% *Aphanizomenon* spp. and 9% *Anabaena* sp.

For the spring diatom experiment microscopy counts (cells per mL) were counted from each culture, which was used to make a mixed diatom community consisting of ~11% *Chaetoceros wighami*, ~18% *Thalassiosira baltica*, ~27% *Skeletonema marinoi*, ~35% *Melosira artica*, and ~9% *Diatoma tenuis* (**Table S1c**).

**Supplementary Table 2.** Amount of delivered read pairs, the number remaining after merging of forward and reverse reads and the final amount of merged reads after quality trimming used to cluster the OTUs. Abbreviations for the diatom experiment are: DNA = Diatoms + No bubbling; NA = No bubbling; DA = Diatoms + bubbling; and A = Bubbling. Abbreviations for the cyanobacteria experiment are: NAC = Cyano + No bubbling; NA = No bubbling; AC = Cyano + Bubbling; and A = Bubbling.

**Supplementary Table 3.** Oxygen measurements in the sediment cores during the autumn and spring incubation experiments. The values are from bottom water overlying the sediment (SD = 1). The experiments are as follows: Bubbling) water phase gently bubbled with air (*n* = 3 to 4); Bubbling + Cyano/Diatom) water phase gently bubbled with air and added cyanobacteria or diatom biomass (*n* = 4); No bubbling) no bubbling, i.e. only air-water interface diffusion of oxygen (*n* = 3); and No bubbling + Cyano/Diatom) no bubbling with added cyanobacteria or diatom biomass (*n* = 4). Values after 9 days in the diatom experiment are for duplicates. The low values for the autumn cyanobacteria experiment at day 0 are because the sediment cores had an unmixed water phase over one night after the sediment had been sampled.

**Supplementary Table 4.** Chemistry measurements in the water and sediment phases from the autumn (Cyanobacteria) and spring (Diatom) experiments, SD = 1. Experiments are as follows: Bubbling: water phase gently bubbled with air (*n* = 3 to 4); Bubbling + Cyano/Diatom: water phase gently bubbled with air and added cyanobacteria or diatom biomass (*n* = 4); No bubbling (*n* = 3); and No bubbling + Cyano/Diatom (*n* = 4). Values after 9 days in the diatom experiment are for duplicates. Ammonium was also measured on field samples and consists of the same amount of replicates except that Cyanobacteria experiment Zero time-point sediment (*n* = 2) and Bubbling + Cyano sediment (*n* = 3).

**Supplementary Table 5.** Shannon H’s diversity indexes of the 16S rRNA gene OTUs in the sediment from the autumn cyanobacteria and spring diatom experiment. Data was subsampled to the lowest sample size and bootstrapped 100 times before analysis (Autumn 4094 counts; and Spring 40044 counts), SD = 1.

**Supplementary Table 6.** Pearson correlations of the top 30 OTUs from the sediment surface of the autumn cyanobacteria and spring diatom experiment. The top figure shows data from the cyanobacteria experiment (addition of cyanobacteria biomass *n* = 12-15 and no cyanobacteria biomass I = 10-13) while the bottom figure shows data with diatom addition (addition of diatom biomass *n* = 8 and no diatom biomass *n* = 7). Chemistry data from all incubation days were used to calculate the correlation.

**Supplementary Table 7.** Mass balance calculations of mol (10^6^) nitrite + nitrate and ammonium in the sediment surface and the water phase at the start and end of the autumn (Cyanobacteria) and spring (Diatom) experiments. Mol were calculated for these compounds to take into account the water volume (that decreased with each subsampling) and the amount of these compounds in the top 1 cm sediment volume. The average of the sediment field NO_2_^-^+NO_3_^-^ and NH_4_^+^ minus the end time points were used to determine the loss of NO_2_^-^+NO_3_^-^ and NH_4_^+^ in the sediment. The increase of NO_2_^-^+NO_3_^-^ in the water column was determined by subtracting the average of the field by the end time points. The ratio of sediment NO_2_^-^+NO_3_^-^ and NH_4_^+^ loss and water NO_2_^-^+NO_3_^-^ increase was used to estimate the % of potential diffusion and nitrification from the sediment.

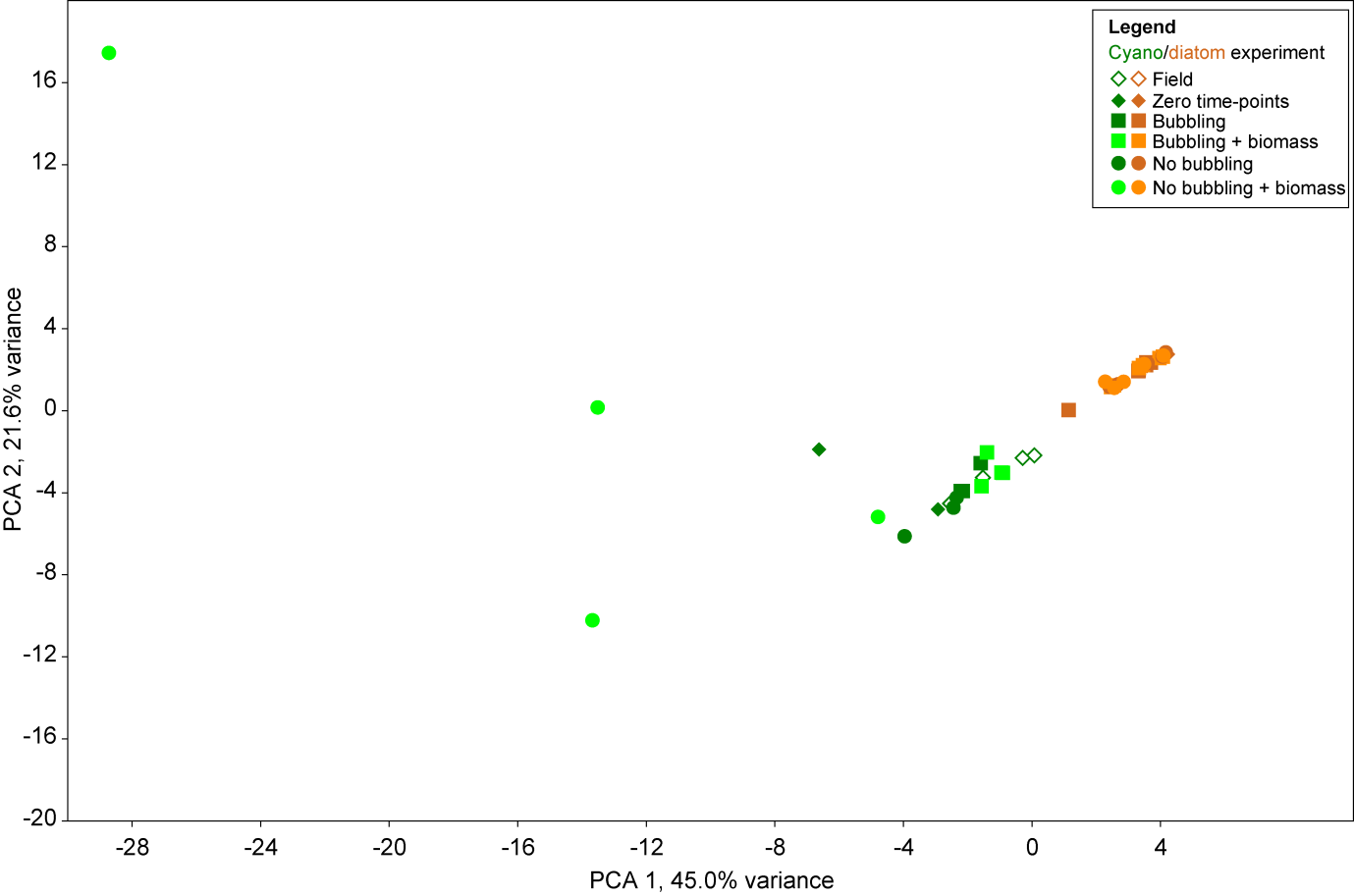


**Supplementary Figure 1.** Principal component analysis based on the 16S rRNA gene data obtained from the sediment surface from the autumn cyanobacteria and spring diatom experiments. OTUs were grouped to the lowest annotated taxonomical level. For the diatom experiment the 0-1 cm and 1-2 cm sediment layers were merged.
